# Supplementary material for: A New Strain of Lecanicillium uredinophilum Isolated from Tibetan Plateau and Its Insecticidal Activity
Source: Microorganisms. 2022 Sep 14;10(9):1832. doi: 10.3390/microorganisms10091832 (PMC9501334; doi:10.3390/microorganisms10091832)
Supplement: Supplementary file 1 [file microorganisms-10-01832-s001.zip › Table S1.pdf]

Supplementary material S1 Gene and primers used in the phylogenetic analyses.

Gene (reference)	Primer	Sequence	PCR condition
ITS	ITS1	TCCGTAGGTGAACCTGCGG	(1) Initialization at for 3 min at 94°C.<br>(2) 40 cycles of denaturation at 94°C for 45 s, annealing at 56 °C for 50 s, and extension at 72°C for 1 min.<br>(3) Final elongation at 72°C for 10 min.<br>(4) Storage at 12°C (Gonalves et al, 2021).
	ITS4	TCCTCCGCTTATTGATATGC	
nrSSU	nrSSU-CoF	TCTCAAAGATTAAGCCATGC	
	nrSSU-CoR	TCACCAACGGAGACCTTG	
nrLSU	LROR	ACCCGCTGAACTTAAGC	
	LR5	TCCTGAGGGAAACTTCG	
RPB_1	RPB1Cr	CCNGCDATNTCRTTRTCCATRTA	(1) Initialization at for 2 min at 94°C.<br>(2) 10 cycles of denaturation at 94°C for 30 s, annealing at 64 °C for 1 min, and extension at 72°C for 1 min.<br>(3) followed by 35 cycles of denaturation at 94°C for 30 s, annealing at 54 °C for 1 min, and extension at 72°C for 1 min.<br>(4) Final elongation at 72°C for 10 min.<br>(5) Storage at 12°C.
	CRPB1A	CAYCCWGGYTTYATCAAGAA	
RPB_2	fRPB2-7cR	CCCATRGCTTGTYYRCCCAT	(1) Initialization at for 3 min at 95°C.<br>(2) 40 cycles of denaturation at 95°C for 1 min, annealing at 52 °C for 2 min, and extension at 72°C for 90 s.<br>(3) Final elongation at 72°C for 10 min.<br>(4) Storage at 12°C.
	fRPB2-5F	GAYGAYMGWGATCAYTTYGG	
