# Supplementary material for: A New Strain of Lecanicillium uredinophilum Isolated from Tibetan Plateau and Its Insecticidal Activity
Source: Microorganisms. 2022 Sep 14;10(9):1832. doi: 10.3390/microorganisms10091832 (PMC9501334; doi:10.3390/microorganisms10091832)
Supplement: Supplementary file 1 [file microorganisms-10-01832-s001.zip › Table S2.pdf]

Supplementary material S2 Specimen information and GenBank accession number for strains used in this study

Species	Voucher information	GenBank accession number				
		nrLSU	*nrSSU*	*RPB_1*	*RPB_2*	*ITS*
Lecanicillium uredinophilum	QHLA	MZ768916	MZ768917	ON107271	ON107270	MZ768909
Lecanicillium acerosum	CBS418.81	KM283786	KM283762	KM283832	KM283852	EF641893
Lecanicillium antillanum	CBS350.85	AF339536	AF339585	DQ522396	DQ522450	AJ292392
Lecanicillium aphanocladii	CBS797.84	KM283787	KM283763	KM283833	KM283853	-
Lecanicillium aphanocladii	GZUIFR-lun1505	MN944423	MN963920	MT006062	MT006067	MN944448
Lecanicillium aranearum	CBS726.73a	AF339537	AF339586	EF468887	EF468934	AJ292464
Lecanicillium araneogenum	GZU1032Lea	KX845704	KX845706	KX845670	KX845702	-
Lecanicillium attenuatum	CBS402.78	AF339565	AF339614	EF468888	EF468935	AJ292434
Lecanicillium attenuatum	GZUIFR-lun1405	MN944422	MN963919	MTT006061	MT006066	MN944447
Lecanicillium attenuatum	KACC42493	KM283780	KM283756	KM283826	KM283846	-
Lecanicillium cauligalbarum	GZUIFR-ZHJ02	MH730664	MH730666	MH801921	MH801923	MH801925
Lecanicillium dimorphum	CBS345.37	KM283788	KM283764	KM283834	KM283854	-
Lecanicillium dimorphum	CBS363.86	AF339559	AF339608	EF468890	-	-
Lecanicillium flavidum	CBS300.70D	KM283789	KM283765	-	KM283855	EF641877
Lecanicillium fungicola var. *aleophilum*	CBS357.80	KM283791	KM283767	KM283335	KM283856	NR111064
Lecanicillium fungicola var. *fungicola*	CBS992.69	KM283792	KM283768	-	KM283857	NR119653
Lecanicillium fusisporum	CBS164.70	KM283793	KM283769	KM283836	KM283858	AJ292428
Lecanicillium lecanii	CBS101247	KM283794	KM283770	KM283337	KM283859	JN049836
Lecanicillium lecanii	CBS101267	KM283795	KM283771	KM283838	KM283860	-
Lecanicillium longisporum	CBS102072	KM283796	KM283772	KM283839	KM283861	-
Lecanicillium longisporum	CBS126.27	KM283797	KM283773	KM283840	KM283862	-
Lecanicillium muscarium	CBS143.62	KM283798	KM283774	KM283841	KM283863	-
Lecanicillium pissodis	CBS118231	KM283799	KM283775	KM283842	KM283864	-
Lecanicillium psalliotae	CBS532.81	AF339560	AF339609	EF469096	EF469112	JN049846
Lecanicillium sabanense	JCHA5	KC875225	KC633251		KC633249	KC633232
Lecanicillium sp.	GZUIFR-lun1403	MN944420	MN963917	MT006059	MT006064	MN944446
Lecanicillium subprimulinum	HKAS99548	MG585315	MG585316	-	-	MG585314
Lecanicillium subprimulinum	HKAS99548	MG585319	MG585320	-	-	MG585318
Lecanicillium tenuioes	CBS309.85	KM283802	KM283778	KM283844	KM283866	JN036556
Lecanicillium uredinophilum	KACC47756	KM283783	KM283759	KM283829	KM283849	-
Lecanicillium wallacei	CBS101237	AY184967	AY184978	EF469102	EF469119	-
Alternaria abundans	CBS534.83	MH873354	MH861640	-	KC584448	MH861639

–: no nitrogen.
